# Supplementary material for: Prolonged Disability following Re-Exposure after Complete Recovery from Aerotoxic Syndrome: A Case Report
Source: Int J Environ Res Public Health. 2023 Dec 8;20(24):7156. doi: 10.3390/ijerph20247156 (PMC10742713; doi:10.3390/ijerph20247156)
Supplement: Supplementary file 1 [file ijerph-20-07156-s001.zip › Supplementary Table S1.pdf]

Supplementary Table S1.

Days post exposure	Exposure + 1 week	5 weeks	10 weeks	4 months and 3 weeks	6 months	Re-exposure +2 weeks	6 weeks	3 months and 2 weeks	5 months	7 months and 1 week	9 months	11 months and 2 weeks
Days post exposure, initial injury	9	35	72	133	168	989	1015	1073	1113	1176	1227	1295
Days post exposure, second injury	N/A	N/A	N/A	N/A	N/A	15	41	99	139	202	253	321
Headache	4	2	2	2	1	3	1	1	1	1	1	2
Pressure in Head	2	1	2	1	1	2	1	0	0	1	1	1
Neck Pain	0	0	0	0	0	0	0	0	0	0	0	0
Nausea or Vomiting	1	0	0	0	0	1	0	0	0	0	0	2
Dizziness	3	1	0	0	0	1	1	0	0	0	0	2
Blurred Vision	4	2	0	0	0	3	2	2	1	1	2	1
Balance Problems	1	1	1	1	1	2	1	1	1	1	1	1
Sensitivity to Light	2	2	2	2	1	1	1	0	0	1	0	0
Sensitivity to Noise	1	2	1	1	0	1	0	0	0	0	0	0
Feeling Slowed Down	4	3	1	2	1	3	2	1	1	2	1	2
Feeling like "in a fog"	4	3	1	1	0	3	2	1	1	1	1	1
Don't feel right	4	3	1	1	0	3	2	1	1	1	1	1
Difficulty Concentrating	4	3	2	2	1	3	2	1	2	2	2	2
Difficulty Remembering	4	3	2	2	1	4	3	2	2	2	2	2
Fatigue or Low Energy	4	2	3	1	1	3	2	1	1	0	1	2
Confusion	4	2	1	1	1	3	2	0	2	1	0	1
Drowsiness	4	1	0	0	0	2	0	0	0	0	0	0
Trouble Falling Asleep (if applicable)	1	1	0	0	0	1	0	0	0	0	0	0
More Emotional	3	3	0	1	1	3	1	2	2	0	1	1
Irritability	3	3	1	1	1	3	2	1	1	1	1	1
Sadness	2	1	0	0	0	2	2	1	1	1	2	2
Nervous or Anxious	4	3	1	0	1	3	2	1	1	1	1	1
Total Number of Symptoms	21	20	14	14	12	21	17	13	14	14	14	17
Symptom Severity Score	63	42	21	19	12	50	29	16	18	17	18	25
